# Steady flow and heat transfer analysis of Phan-Thein-Tanner fluid in double-layer optical fiber coating analysis with Slip Conditions

**DOI:** 10.1038/srep34593

**Published:** 2016-10-06

**Authors:** Zeeshan Khan, Rehan Ali Shah, Saeed Islam, Bilal Jan, Muhammad Imran, Farisa Tahir

**Affiliations:** 1 Department of Mathematics, Abdul Wali Khan University, Mardan, KPK, Pakistan; 2 Department of Computer Science and IT, Sarhad University of Science and IT, KPK, Pakistan; 3 Department of Mathematics, University of Engineering and Technology, Peshawar, KPK, Pakistan; 4 Department of Physics, Abdul Wali Khan University, Mardan, KPK, Pakistan

## Abstract

Modern optical fibers require double-layer coating on the glass fiber to provide protection from signal attenuation and mechanical damage. The most important plastic resins used in wires and optical fibers are plastic polyvinyl chloride (PVC) and low-high density polyethylene (LDPE/HDPE), nylon and Polysulfone. In this paper, double-layer optical fiber coating is performed using melt polymer satisfying PTT fluid model in a pressure type die using wet-on-wet coating process. The assumption of fully developed flow of Phan-Thien-Tanner (PTT) fluid model, two-layer liquid flows of an immiscible fluid is modeled in an annular die, where the fiber is dragged at a higher speed. The equations characterizing the flow and heat transfer phenomena are solved exactly and the effects of emerging parameters (Deborah and slip parameters, characteristic velocity, radii ratio and Brinkman numbers on the axial velocity, flow rate, thickness of coated fiber optics, and temperature distribution) are reported in graphs. It is shown that an increase in the non-Newtonian parameters increase the velocity in the absence or presence of slip parameters which coincides with related work. The comparison is done with experimental work by taking *λ* → 0 (non-Newtonian parameter).

Investigating the behavior of the boundary layer of a viscoelastic fluid, on a continuous surface stretching, is important for the analysis of polymer extrusion, drawing of plastic films, fiber optics and wires. The significance in industrial process applications has led to a deep interest by researchers for the study of viscoelastic fluid flow and heat transfer in fiber and wire coating process. The optical fiber coating is an industrial process for provision of insulation, environmental safety, mechanical damage and guard against signal attenuation. The simple and suitable process for wire coating is the coaxial extrusion process that operates at the maximum pressure, temperature and wire drawing speed. This coating of the continuum velocity and melt-polymer produces high pressure in a particular region which results into strong bonding and fast coating. Many researches such as Han and Rao^1^, Nayal^2^, Caswell^3^ and Ticker^4^ studied the co-extrusion process in which either the polymer is extruded on axially moving belt or the fiber (wire) is dragged inside a die filled with molten polymer.

The manufacturing of optical fibers is a series of automated inline process such as the drawing of glass fiber from a softened Silica preform in draw furnace, the coaling of freshly drawn glass fiber in helium injected Coaling System, and the double layer coating of polymers on glass fibers. Then, the optical fiber manufacturing becomes complete as the liquid fiber coatings are cured by Ultraviolet (UV) Lamps.

The coatings are necessary to provide mechanical protection and to prevent the ingress of moisture into microscopic flaws on the fiber surface. The optical fibers today in general are characterized by a double-layer coating structure: an inner layer (called a primary coating layer) made of soft coating material and an outer layer (called a secondary coating layer) made of hard coating material. The role of the Primary layer is to minimize attenuation due to micro bending, while the secondary layer protects the primary coating against mechanical damage. The widespread industrial success of optical fibers as a practical alternative to copper wiring could be attributed to these UV-curable coatings.

Two types of coating process are used for double-layer optical fiber coating, while being pulled at high speed, wet-on-dry (WOD) and wet-on-wet (WOW) process. In wet-on-dry coating process, the glass fiber passes through a primary coating applicator which is immediately cured by UV lamps, and then the fiber enters a secondary coating applicator, again followed by UV lamps. However, in the WOW coating process, the glass fiber passes through both the primary and secondary coating applicators and then both these coatings are cured by UV lamps. In the past, the majority of optical fiber drawing systems used the wet-on-dry process, but recently the wet-on-wet coating process has gained significant popularity in optical fiber manufacturing industry. Here, in this study, we also applied the wet-on-wet coating process for optical fiber coating as shown in Fig. 1.

In fiber coating, the fiber drawing velocity and the quality of material are more important. And after leaving the die, the temperature of the coating material is also important.

Different types of fluids are used for wire and fiber optics coating, which depends upon the geometry of die, fluid viscosity, temperature of the wire or fiber optics and the molten polymer. Most relevant work on the wire and fiber optics coating are thus summarized in the following.

The power law fluid model was used by Akter and Hashmi^5^,^6^ for wire coating. Siddiqui *et al*.^7^ used third grade fluid for wire extrusion in a pressurized die. Fenner and Williams^8^ investigated the flow in the tapering section of a pressurized die. Unsteady second grade fluid with oscillating boundary condition inside the wire coating die was investigated by Shah *et al*.^9^. Exact solution was obtained for unsteady second grad fluid in wire coating analysis^10^. Oldroyd 8-constant fluid was used for wire coating analysis by Shah *et al*.^11^. Majid *et al*.^12^ studied wire coating using MHD Oldroyd 8-constant fluid. Shah *et al*.^13^ studied third grade fluid with heat transfer in the wire coating analysis.

The interest in heat transfer problems involving non-Newtonian fluids have grown considerably as the application of non-Newtonian fluids perpetuates through various industries, including polymer processing and electronic packaging. Heat transfer analysis is very important for the advancement of science and technology, modern instruments such as micro-electro-mechanical systems (MEMS), laser coolant lines and compact heat exchangers are being used for many purposes. Laminar heating and cooling occur an increasing variety in such instruments. Consequently, the results for the flows and heat transfer of non-Newtonian fluids are needed. A complete survey of the literature is impractical. However, a few studies are listed here to provide starting points for a broader literature search. Shah *et al*.^14^ studied the wire coating analysis with linearly varying temperature. Mitsoulis^15^ studied the wire coating flow with heat transfer. The corresponding heat transfer problem of fully developed pipe and channel flows of PTT fluid was also investigated by Oliveira and Pinho^16^.

Recently, a viscoelastic fluid model known as Phan-Thien-Tanner (PTT) model is widely used for wire and fiber coating^17^. It is a nonlinear viscoelastic model which incorporates shear thinning, shear viscosity, normal stress difference and an elongation parameter which reproduces many of the characteristics of the rheology of polymer solutions and other non-Newtonian fluids. Many researchers studied the post-treatment analysis of wire coating with heat transfer^18^. Wagner and Mitsoulis^19^ investigated the wire coating with the effect of die design. Numerical solution for wire coating analysis using a Newtonian fluid was investigated by Bagley and Storey^20^. Oliveira and Pinho^21^ studied the problem of fully developed channel and pipe flows of PTT fluids and obtained an analytical expression for velocity fields and stress components in both geometries.

A survey of literature indicates that much attention is given to slip effect, especially from polymer industry (polymer melts), which exhibits a macroscopic wall slip. It ranges from technological application to medical application, especially in polishing artificial heart valves and also used for wire and fiber coating. Being inspired from such practical applications, several authors discussed the slip effect on fluid flow. Hayat *et al*.^22^ and Asghar *et al*.^23^ discuss the effects of slip condition on third order fluid. Ellahi^24^ discuss the slip condition of an Oldroyd 8- constant fluid and Sajid *et al*.^24^,^25^ investigate the effect of slip condition on thin film flow. The influence of slip conditions on the thin film flow of a third order fluid was investigated by Nargis and Tahir^26^. Asghar *et al*.^27^ studied the effects of partial slip on flow of a third grade fluid. Recently, Rehan *et al*.^28^ studied wire coating for heat transfer flow of a viscoelastic PTT fluid with slip boundary conditions. Recently, Hatzikiriakos^29^ discussed the wall slip of molten polymer. Simulation of coating flows with slip effects by Ngamaramvaranggul *et al*.^30^. Ferras *et al*.^31^ investigated an analytical solutions for channel flows of Phan-Thien-Tanner and Giesekus fluids under slip. The same author studied the annular flow of viscoelastic fluids using numerical and analytical technique^32^. Georgiou *et al*.^33^ also investigated slip yield stress effects in start-up Newtonian Poiseuille flows.

All these attempts were related to a single layer coating flow. On double-layer coating flow there are also few investigations.

Immiscible fluid flow is used for many industrial and manufacturing processes such as oil industry or polymer production. Kim *et al*.^34^ examined the theoretically prediction on the double-layer coating in wet-on-wet optical fiber coating process. Double-layer coating liquid flows were used by Kim *et al*.^35^ in optical fiber manufacturing. For this purpose power-law fluid model was used. Recently Zeeshan *et al*.^36^ used Phan-Thien-Tanner fluid in double-layer optical fiber coating. The same author^37^ investigated double-layer resin coating of optical fiber glass using wet-on-wet coating process with constant pressure gradient. Two-phase flow of an Oldroyd 8-constant fluid was used for optical fiber coating by Zeeshan *et al*.^38^. Flow and heat transfer of two immiscible fluids in double-layer optical fiber coating is investigated by Zeeshan *et al*.^39^.

Zeeshan *et al*.^36^,^37^ has considered PTT fluid as coating material in double-layer optical fiber coating analysis and studied the effect of emerging parameters. However, they do not investigate the effect of slip conditions in their study. The aim of the present study is to analyze the double-layer optical fiber coating using viscoelastic PTT fluid with slip condition in wet-on-wet coating process. To the best of my knowledge, no one has investigated the double-layer coating in wet-on-wet coating process for optical fiber coating using as coating material modeled as Phan-Thien-Tanner fluid such as a melt polymer with slip boundary conditions. The equations characterizing the flow and heat transfer phenomena are solved exactly and the effects of emerging parameters are shown with the help of graphs. To the best of my knowledge, no such analysis of the double-layer coating flows of PTT fluid using slip conditions is available in the literature.

Additionally, at the end the result of the present work is also compared with the experimental results already published^34^ by taking *λ* → 0 (non-Newtonian parameter).

## Modeling of the problem

The schematic diagram of two-phase flow model in a pressurized coating die of length *L* is shown in Fig. 2. The die and fiber are concentric. The coordinate system is taken at the center of the optical fiber, in which *r* is taken perpendicular to the flow direction *z*. The coating process is performed in two phases. In the first phase the uncovered fiberglass of radius *R*_*w*_ is dragged with constant velocity *U* into the primary coating liquid. In the second phase the wet coating passes through the secondary coating die of the radius of radius *R*_*d*_. This way the fiber leaves the system with two layers of coating. The wet layers are dried up by ultraviolet (UV) lamps. The flow is considered steady, laminar and axisymmteric.

The design of fiber coating dies is of primary importance since it significantly affects the quality of the final product. Here, a pressure type die is considered because within this die melt meets the optical fiber where a complex flow field exists and its surrounding is necessary for the design of better dies with optimum performance.

Sip boundary conditions are subjected at the moving optical fiber and the stationary die wall in the die design. The liquid parameters at each phase are generalized by corresponding phase number denoted by *j*(*j* = 1, 2).

The basic equations governing the flow of incompressible fluids are:


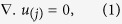



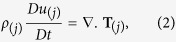



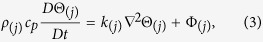



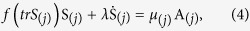


where *ρ*_(*j*)_, density of the fluid, T_(*j*)_, shear stress tensor, *c*_*p*_, the specific heat, *D*/*Dt*, denotes the material derivative, *k*_(*j*)_, the thermal conductivity,Θ_(*j*)_, the fluid temperature, Φ_(*j*)_ the dissipation function, *trS*_(*j*)_, the trace of extra stress tensor, 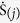, upper contra-variant convicted tensor, μ_(*j*)_, the viscosity of the fluid and A_(*j*)_ is the deformation rate tensor.

The shear stress tensor given in Eq. (2) and deformation rate tensor given in Eq. (4) is defined as:


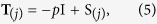



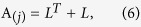


where I, is the identity tensor and the superscript, *T*, stands for the transpose of a matrix and *L* = ∇*u*_(*j*)_.

The upper contra-variant convicted tensor 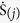 in Eq. (4) is given by


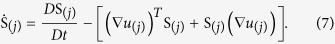


The function 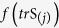 is given by Tanner^28^,^40^,^41^


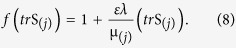


In Eq. (8), *f*(*tr*S_(*j*)_) is the stress function in which *ε* is related to the elongation behavior of the fluid. For *ε* = 0, the model reduces to the well-known Maxwell model and for *λ* = 0, the model reduces to Newtonian one.

With the above frame of reference and assumptions the fluid velocity, extra stress tensor and temperature filed are considered as


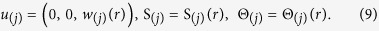


Using assumptions and Eq. (9), the continuity Eq. (1) satisfied identically and from Eqs (2,

we arrive at:


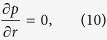



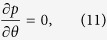



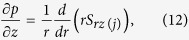



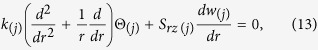



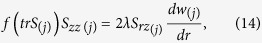



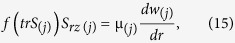



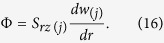


From Eqs (10) and (11), it is concluded that *p* is a function of *z* only. Assuming that the pressure gradient along the axial direction is constant. Thus we have *dp*/*dz* = Γ
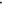


Integrating Eq. (12) with respect to *r*, we get


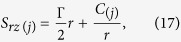


where *C*_(*j*)_ is an arbitrary constant of integration.

By substituting Eq. (17) in Eq. (15), we have


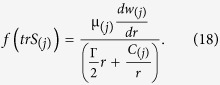


Combining Eqs (14), (15) and (17), we obtain the explicit expression for a normal stress component *S*_*zz*_ as:


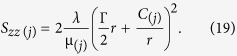


From Eqs (8) and (18), we have


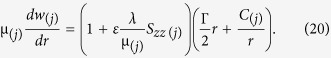


Inserting Eq. (19) in Eq. (20), we obtain an analytical expression for axial velocity as:


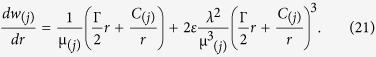


And the temperature distribution is


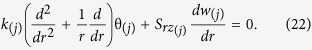


**Figure 1:**
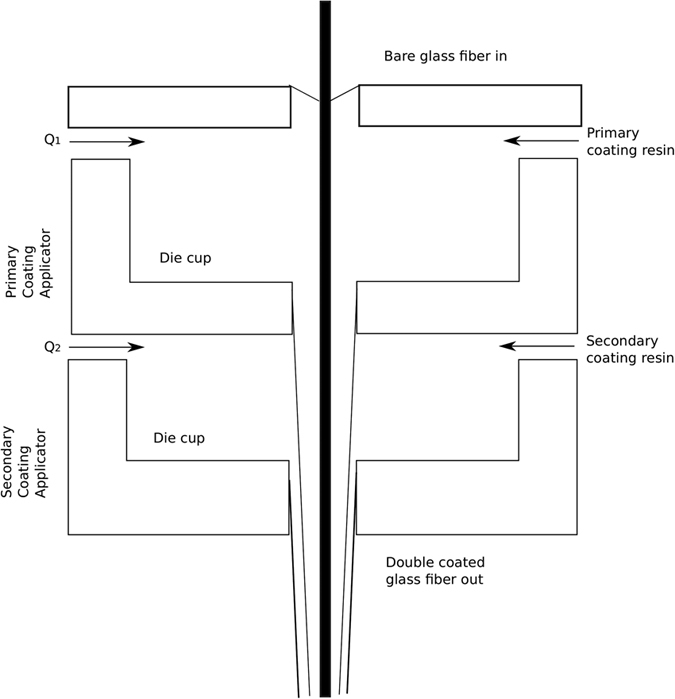
Double-layer optical fiber coating in wet-on-wet coating process.

**Figure 2:**
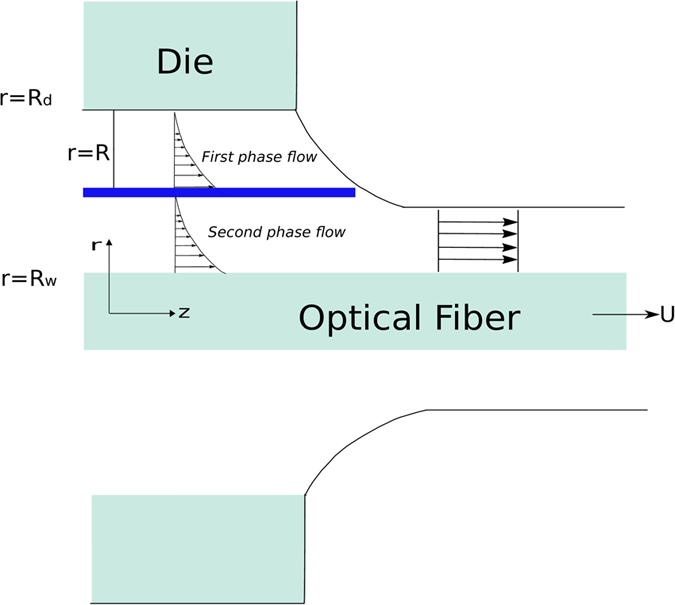
Two-phase flow model in coating die.

The boundary conditions on *w*_(*j*)_ are slip conditions, and the boundary condition on θ_(*j*)_ are 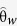 at the fiber optics and 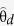 at the die wall. For the problem displayed in Fig. 1, at the fluid interface, we utilize the assumptions that the velocity, the shear stress, and the pressure gradient along the flow direction and the temperature and the heat flux are continuous, which are given as follows.

The relevant boundary and interface conditions^34^,^35^,^36^,^37^,^38^,^39^ on the velocity are


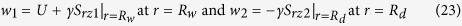



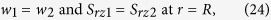


The relevant boundary and interface conditions^36^,^37^,^38^,^39^ on the temperature are


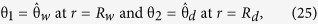



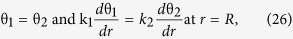


We introduce the non-dimensional flow variables as


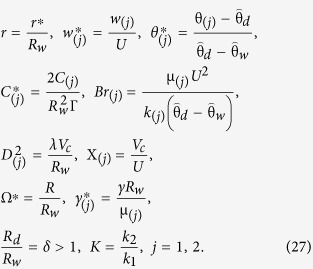



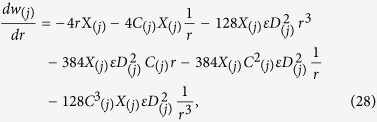



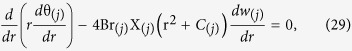



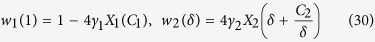



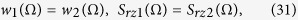



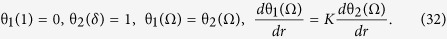


where 
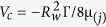
 is the characteristic velocity scale, 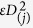 is the characteristic Deborah number based on velocity scale *V*_*c*_, X_(*j*)_ has physical meaning of a non-dimensional pressure gradient and Br_(*j*)_ is the Brinkman number and *j* = 1, 2 stands for primary and secondary coating layer flows respectively.

## Solution of the problem

To obtain the solution for the velocity field and temperature distribution for both layers, we solve Equations (28) and (29), corresponding to the boundary conditions given by equations (30)–(32), 45, 46 respectively.

Primary layer velocity field, flow rate, thickness of the coated fiber optics and temperature are


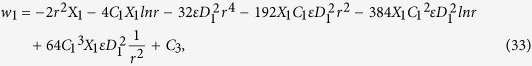



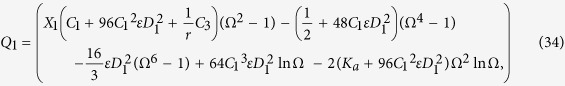



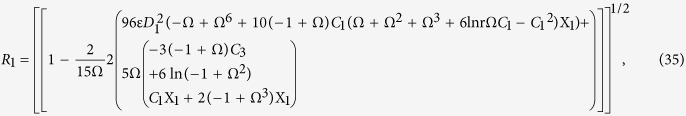



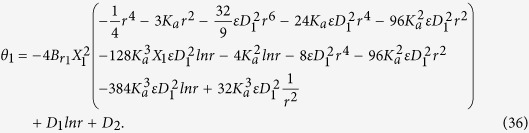


Secondary layer velocity field, flow rate, thickness of the coated fiber optics and temperature are


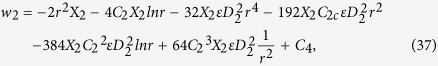



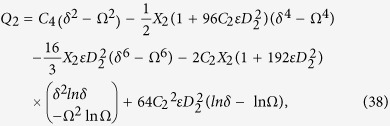



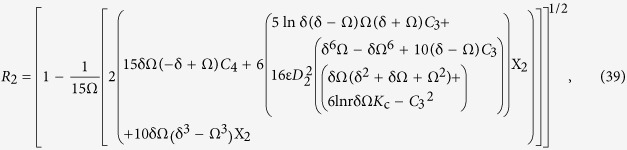



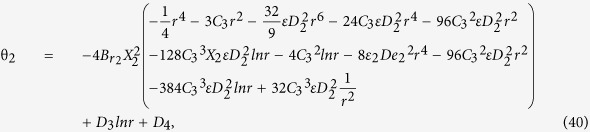


where 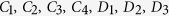 and *D*_4_ are all constants given below:


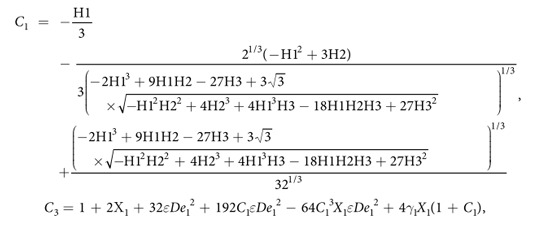



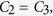



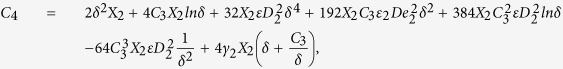



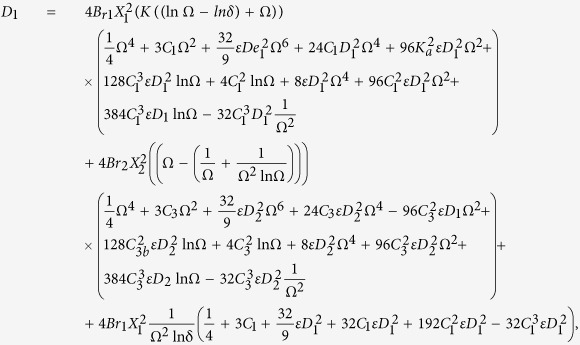



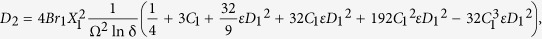



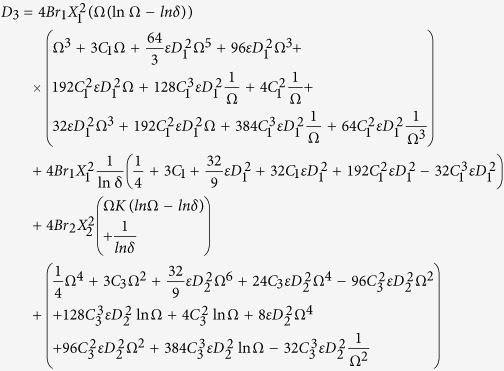



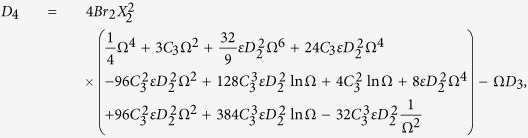


where


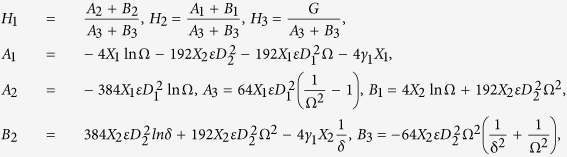



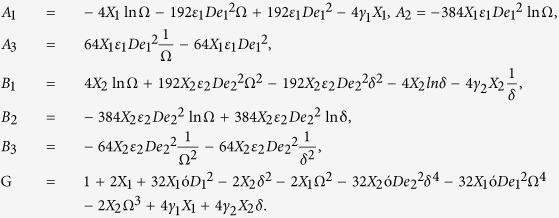


## Results and Discussion

Equations (28) and (29) along with boundary conditions given by Eqs (30)–(32), 45, 46 are solved exactly for the velocity field and temperature distributions, for the primary and secondary layer, related to double-layer optical fiber coating process using melt polymer satisfying Phan-Thien-Tanner (PTT) fluid model in a pressure type die. Wet-on-wet coating process is applied for double-layer optical fiber coating.

The effects of different pertinent parameters such as, slip parameters, Deborah numbers 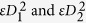, velocity ratio (ratio between the pressure drop and the speed of the wire i.e., 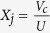), radii ratio *δ*, and the Brinkman numbers *Br*_1_ and *Br*_2_ are discussed and sketched in Figs 3, 4, 5, 6, 7, 8, 9, 10, 11, 12, 13 and 14.

**Figure 3:**
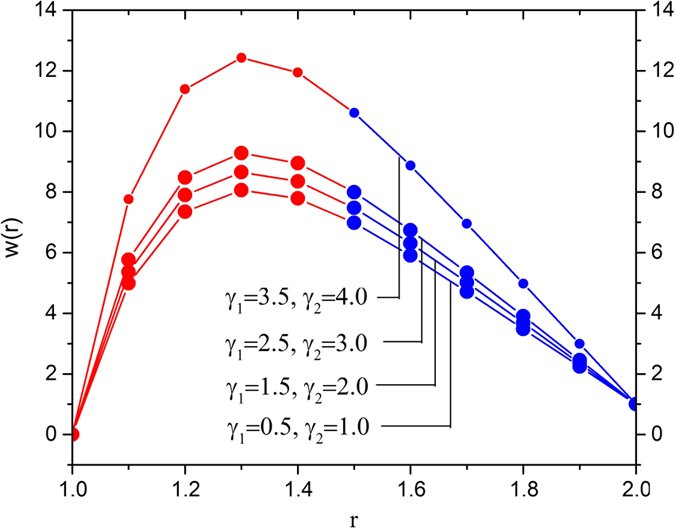
Variation in the velocity field for various values of slip parameters *γ*_1_ and *γ*_2_, fixing 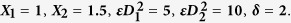

Figure 3 delineates the impact of slip parameters on the velocity field. It is observed that the slip parameters accelerate the melt polymer inside the die. This observation shows the effect of slip parameters is to enhance the velocity field near the surface of the optical fiber in the region 1 < *r* < 1.4 but thereafter, the reverse eeffect is observed.

**Figure 4:**
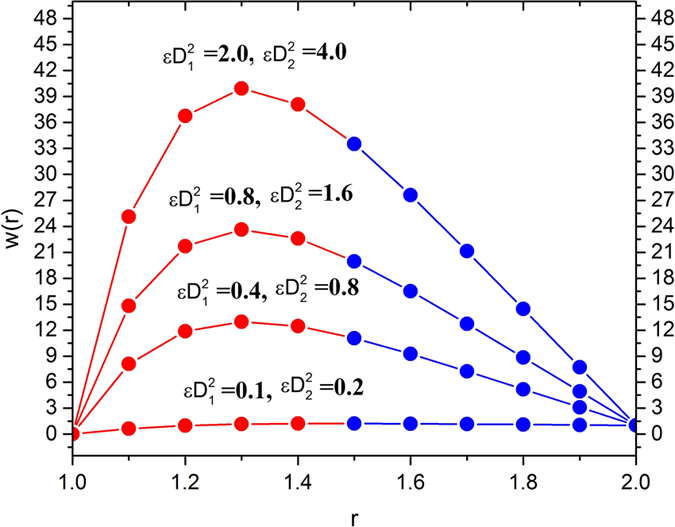
Variation in the velocity field for various values of Deborah numbers 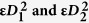, fixing *X* = 1, *X*_2_ = 1.5, γ_1_ = 0.2, γ_2_ = 0.3, *γ* = 2.

**Figure 5:**
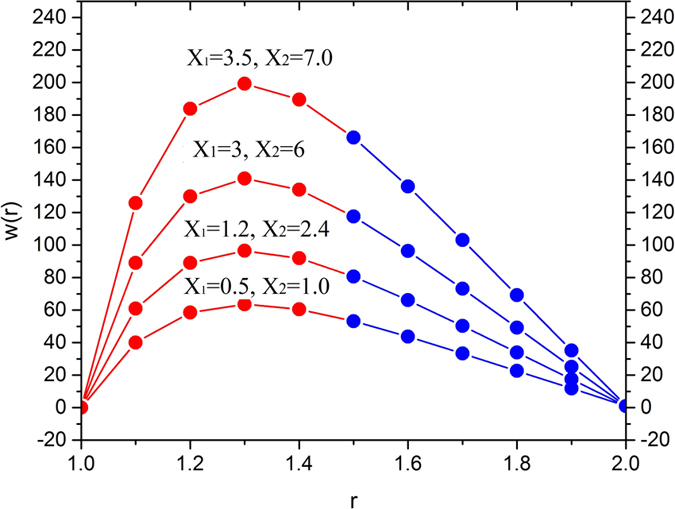
Variation in the velocity field for various values of Deborah numbers X_1_ and X_2_, fixing 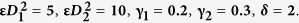

Figures 4 and 5 depict the velocity variation for different values of Deborah numbers and velocity ratio respectively. We observe that the velocity field increases with an increase in these parameters. It is remarkable to note the main contribution on the velocity field is seen in Fig. 5, when the velocity ratio is higher. Also, for low elasticity 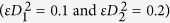, the velocity field deviation slightly differs from Newtonian one, but an increasing 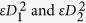, the velocity profiles become more flattered representing the effects of shear thinning, as shown in Fig. 4.

It is interesting to note that an increase in non-Newtonian parameters and slip parameters leads to increase the velocity at all points of the flow domain. As the velocity of coating fluid is an important design requirement, slip parameters and non-Newtonian characteristics of fluid may be used as controlling devices for the required quality.

**Figure 6:**
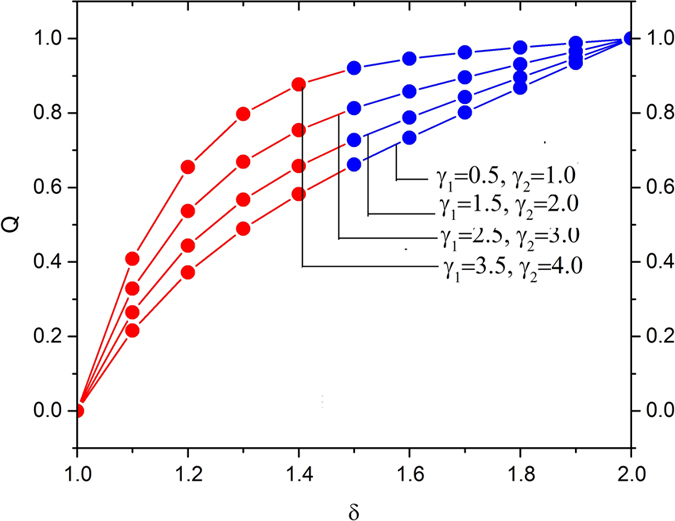
Variation in the volume flow rate for various values of slip parameters *γ*_1_ and *γ*_2_, fixing 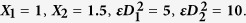

**Figure 7:**
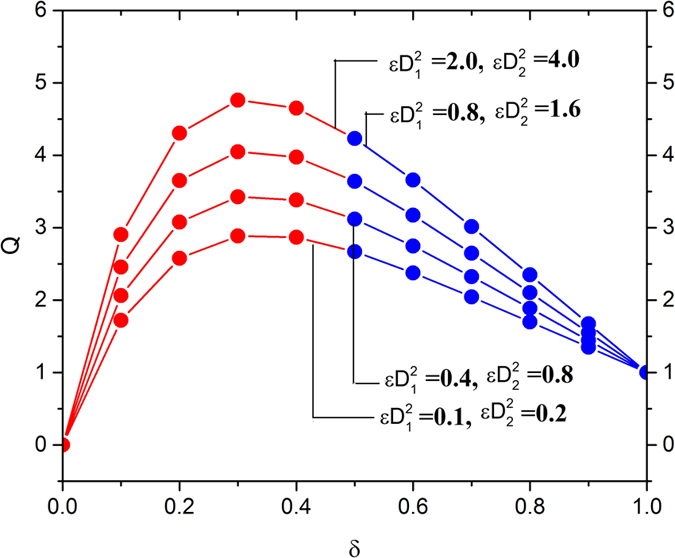
Variation in the volume flow rate for various values of Deborah numbers 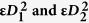 fixing 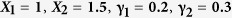.

The effects of slip parameter and Deborah numbers on the volume flow rate along with increasing the radii ratio is shown in Figs 6 and 7. From Fig. 6, it is found that the at higher value of the radii ratio, the effect of slip parameters on the volume flow rate is much more sensitive. It is also evident that increase in Deborah numbers accelerates the volume flow rate in the region 1 < *r* < 1.4, and reverse effects observed afterwards as shown in Fig. 7.

**Figure 8:**
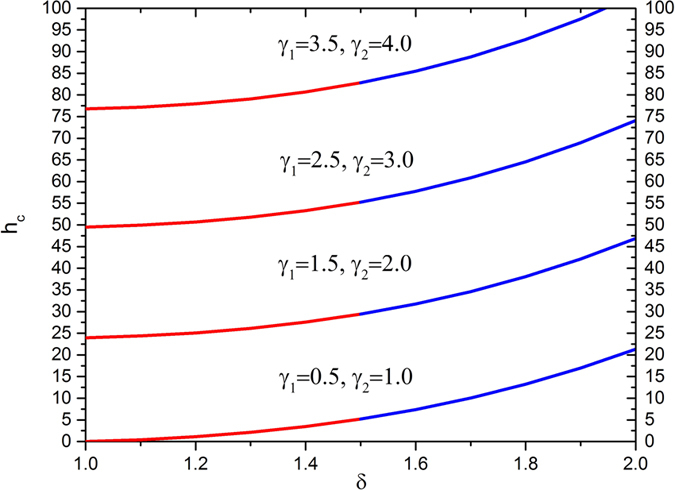
Thickness of coated fiber optics for various values of slip parameters *γ*_1_ and *γ*_2_, fixing 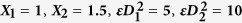.

Figure 8 delineates the effects of enlarging the slip parameters along with an increasing radii ratio on the thickness of coated fiber optics. In this analysis, the Deborah number is set as 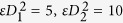 and the velocity ratio at 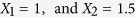. It reveals that for fixed values of these parameters, the thickness of the coated fiber optics increases with an increase in slip parameters as well as radii ratio.

**Figure 9:**
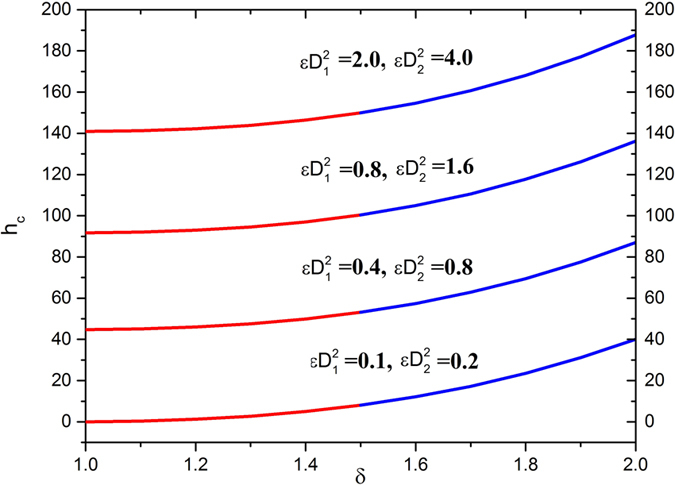
Thickness of coated fiber optics for various values of Deborah numbers 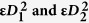 fixing 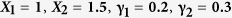.

Figure 9 shows the effects on the thickness of coated fiber optics by changing the Deborah numbers and the radii ratio. This indicates that the thickness of the coated fiber optics increases with increases these parameters.

**Figure 10:**
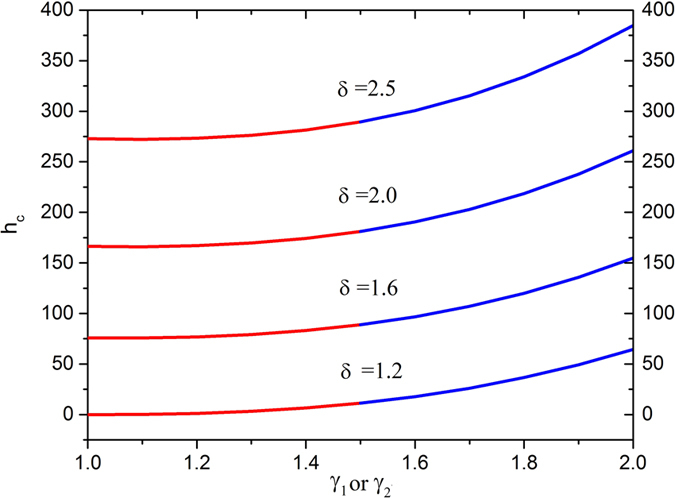
Thickness of coated fiber optics for various values of radii ratio *δ* fixing 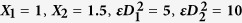.

Figure 10 is sketched to see the effects of radii ratio along the with increasing the slip parameters. In both cases, the thickness of the coated fiber optics tends to increase drastically as the level of radii ratio and the slip parameter increase.

Thus, it is concluded form Figs 8, 9, 10 that the slip parameters, Deborah numbers and the radii ratio contribute to enhance the thickness of the coated fiber optics, so, we may use these parameters to control the thickness of coated fiber optic as a controlling device for the required quality.

**Figure 11:**
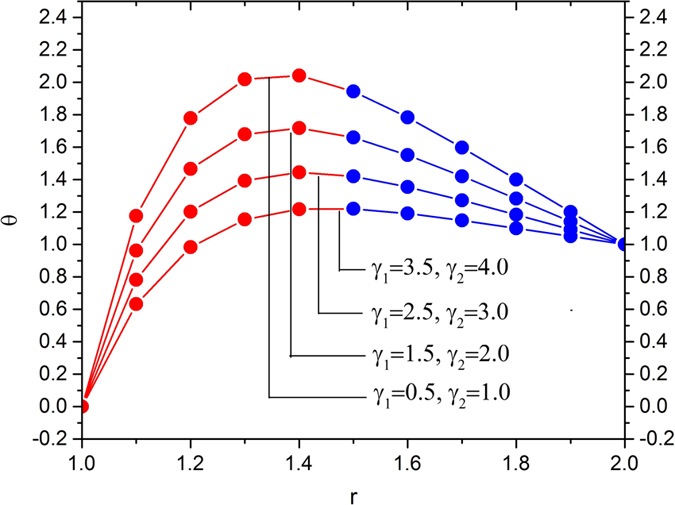
Temperature variation for various values of slip parameters *γ*_1_ and *γ*_2_, fixing 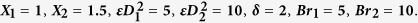

**Figure 12:**
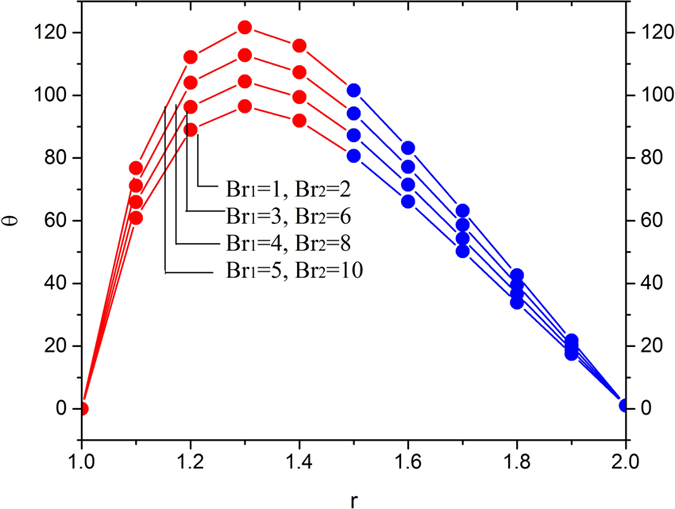
Temperature variation for various values of Brinkman number *Br*_1_ and Br_2_, fixing 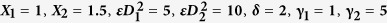.

**Figure 13:**
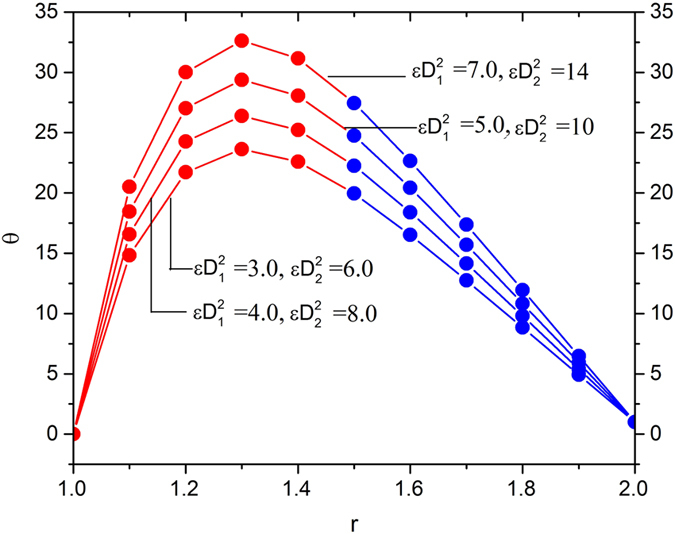
Temperature variation for various values of Deborah number parameters 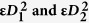, fixing 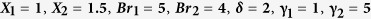.

**Figure 14:**
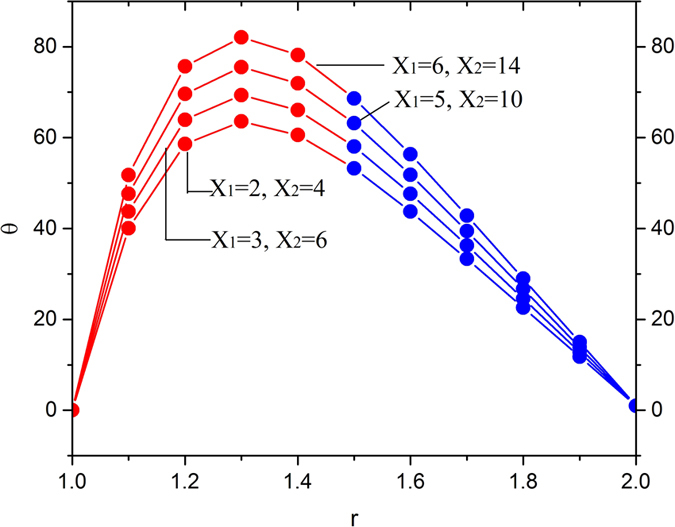
Temperature variation for various values of dimensionless parameters X_1_ and X_2_, fixing 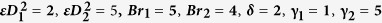.

Figures 11, 12, 13 and 14 display the temperature distribution showing the effects of slip parameters, Brinkman numbers, Deborah numbers and the velocity ratio.

Figure 11 shows the effects of slip parameters on the temperature distribution. In this analysis, we vary the slip parameters, i.e., γ_1_ = 0.5, 1.5, 2.5, 3.5 and γ_2_ = 1, 2, 3, 4, and fixed the other parameters at the reference values, i.e., 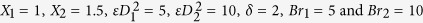. It reveals that the temperature distribution decreases with increases slip parameters. This variation in temperature distribution is higher in the region 1 < *r* < 1.4.

Figures 12 and 13 depict the temperature distribution by showing the effects of Brinkman numbers and Deborah numbers. The effects of Brinkman numbers and Deborah numbers are, to increase the temperature in the region 1 < *r* < 1.4 in all cases, and then, the reverse effect is observed. Thus, it is concluded that viscous heating (*Br*_1_ and *Br*_2_) and non-Newtonian property of melt polymer is favorable in escalating the fluid temperature inside the die near the surface of the optical fiber and it is counterproductive near the inner surface of the die.

Figure 14 depicts the temperature distribution showing the effects of velocity ratio. The point of thermo-transition occurs in the middle of the annular zone. Thus, it is concluded that the velocity ratio enhance the temperature inside the melt polymer used as a coating material near the surface of the optical fiber then it decreases in the region 1.4 < *r* < 2.

At the end the result of the present work is also compared with the experimental results already published^34^ by taking *λ* → 0 (non-Newtonian parameter) which is given in Table 1.

## Conclusion

Exact solutions are obtained for the velocity field and temperature distributions, related to double-layer optical fiber coating process using melt polymer satisfying Phan-Thien-Tanner (PTT) fluid model in a pressure type die. Wet-on-wet coating process is applied for double-layer optical fiber coating. The effects of different pertinent parameters such as, slip parameters, Deborah numbers 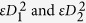, velocity ratio (ratio between the pressure drop and the speed of the wire i.e., 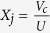), radii ratio *δ*, and the Brinkman numbers *Br*_1_ and *Br*_2_ are investigated. The slip parameters, Deborah numbers and velocity ratio enhance the fluid velocity of the first layer in all cases, and then, the reverse effect is observed. Thus, it is concluded that non-Newtonian property of melt polymer is favorable in escalating the fluid velocity inside the die near the surface of the optical fiber and it is counterproductive near the inner surface of the die. Also, the slip parameters, Deborah numbers and the radii ratio contribute to enhance the thickness of the coated fiber optics, thus, we may use these parameters to control the thickness of coated fiber optics as a controlling device for the required quality.

Furthermore, the effects of Deborah numbers, velocity ratio and Brinkman numbers increase the temperature in the region 1 < *r* < 1.4 in all cases, and then the reverse effect is observed. The effects of slip parameters are quite opposite to that of Brinkman numbers. Thus, it is concluded that viscous heating (*Br*_1_ and *Br*_2_) and non-Newtonian property of melt polymer is favorable in escalating the fluid temperature in the layers near the surface of the optical fiber and having a transition in the middle of the annular region. Also, it reduces to Maxwell and linear viscous model by setting *ε* and *λ* equal to zero, respectively.

At the end the present work is also compared with previously published experimental.

## Additional Information

**How to cite this article**: Khan, Z. *et al*. Steady flow and heat transfer analysis of Phan-Thien-Tanner fluid in double-layer optical fiber coating analysis with Slip Conditions. *Sci. Rep.* **6**, 34593; doi: 10.1038/srep34593 (2016).

## Figures and Tables

**Table 1:** Comparison of present and published work^34^ for Γ = 10, *δ* = 2, γ_1_ = 0.1, γ_2_ = 0.3.

*r*	Present work	Published work	Absolute error
0	1	1	0
0.1	0.81048	0.81048	0
0.2	0.671454	0.671454	0
0.3	0.571454	0.571454	0
0.4	0.492171	0.492171	0
0.5	0.428140	0.428141	0.000001
0.6	0.374170	0.374170	0
0.7	0.326601	0.326601	0
0.8	0.282880	0.282821	1.231 × 10^−32^
0.9	0.241129	0.241129	0
1	0.002000	0.002000	0
